# Impacts of Maternal Nutrition on Sow Performance and Potential Positive Effects on Piglet Performance

**DOI:** 10.3390/ani14131858

**Published:** 2024-06-23

**Authors:** Alexa Gormley, Ki Beom Jang, Yesid Garavito-Duarte, Zixiao Deng, Sung Woo Kim

**Affiliations:** Department of Animal Science, North Carolina State University, Raleigh, NC 27695, USA; agormle@ncsu.edu (A.G.); kjang@ncsu.edu (K.B.J.); yrgaravi@ncsu.edu (Y.G.-D.); zdeng7@ncsu.edu (Z.D.)

**Keywords:** bioactive compounds, highly prolific sows, nutrient requirements, nutritional intervention, oxidative stress, sow productivity

## Abstract

**Simple Summary:**

Modern sows face increased nutritional challenges due to inadequate feeding programs causing excessive maternal tissue loss and reproductive failure. Rapid genetic improvements in reproductive performance should be nutritionally supported; however, current feeding programs have limitations to support the needs of modern sows. Litter size at birth and the birthweight of piglets have increased, increasing the nutrient needs for sows during gestation and lactation. This review also addresses physiological challenges facing modern sows including high oxidative stress, pelvic organ prolapse, and lameness as well as negative impacts on colostrum and milk quality. To mitigate these challenges, there is growing interest in investigating the functional roles of select bioactive compounds as feed additives. Such bioactive compounds have been utilized to reduce disease and illness related to physical stressors, improve colostrum and milk quality, and support sow intestinal health. This review demonstrates that the feeding of modern sows poses unique challenges for nutritionists and the rapid genetic improvements to reproductive performance warrants updated feeding programs and selective use of bioactive compounds.

**Abstract:**

The objectives of this review are to identify the nutritional challenges faced by modern sows and present potential solutions to mitigate excessive maternal tissue loss and reproductive failure as it relates to recent genetic improvements. Current feeding programs have limitations to support the rapid genetic improvements in reproductive performance for modern sows. Since 2012, both litter size at birth and fetal weight have increased by 2.26 pigs per litter and 0.22 kg per piglet, respectively, thereby increasing the nutrient needs for sows during gestation and lactation. Prediction models generated in this review predict that modern sows would need 31% more lysine during gestation when compared with current feeding programs. Physiological challenges facing modern sows are also addressed in this review. High oxidative stress, pelvic organ prolapse, and lameness can directly affect the sow, whereas these physiological challenges can have negative impacts on colostrum and milk quality. In response, there is growing interest in investigating the functional roles of select bioactive compounds as feed additives to mitigate the severity of these challenges. Selenium sources, catechins, and select plant extracts have been utilized to reduce oxidative stress, calcium chloride and phytase have been used to mitigate pelvic organ prolapse and lameness, algae and yeast derivatives have been used to improve colostrum and milk quality, and fiber sources and probiotics have been commonly utilized to improve sow intestinal health. Collectively, this review demonstrates the unique challenges associated with managing the feeding programs for modern sows and the opportunities for revision of the amino acid requirements as well as the use of select bioactive compounds to improve reproductive performance.

## 1. Introduction

**Figure 1:**
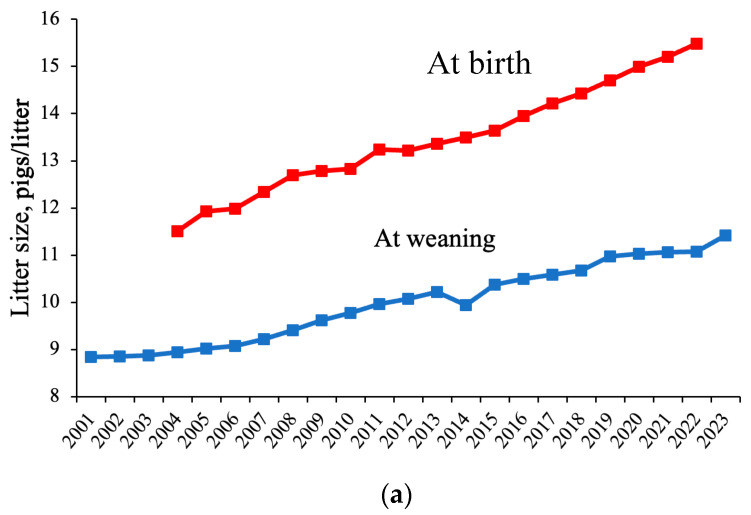

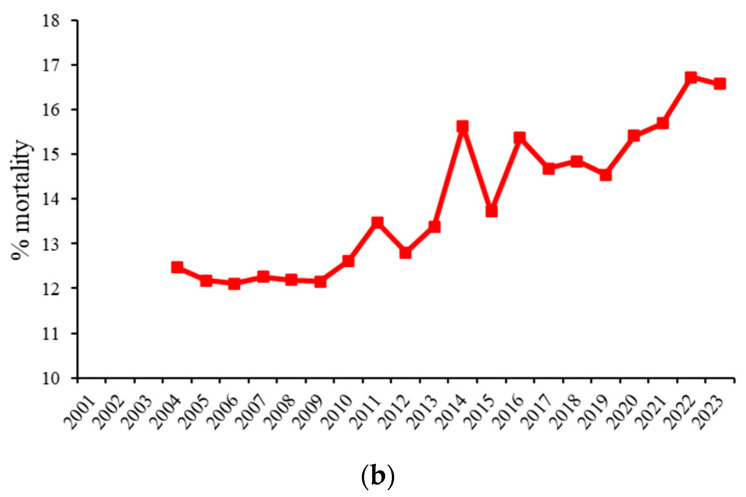
(**a**) Increase in litter size at birth in the United States from 2004 to 2022 [3] and increase in litter size at weaning in the United States per year from 2001 to 2023 [4]. The decline observed in litter size at weaning in 2014 can be attributed to the outbreak of porcine epidemic diarrhea (PED) virus in the US [16]. (**b**) Percent of preweaning mortality in the United States from 2004 to 2023 of piglets born alive [3].

Over the last several decades, genetic improvements have increased litter size and milk yield of sows [1]. Kim et al. [2] reported that since 1935, litter size had increased by three pigs per litter, the average fetus is 40% heavier, and milk production has increased 4-fold by 2013. According to PigCHAMP survey data, the litter size at birth in the United States has increased at a rate of approximately 0.20 pigs per litter per year since 2004 (Figure 1a) [3]. Additionally, the United States Department of Agriculture (USDA) reports that the weaned litter size in the United States has increased at a rate of approximately 0.12 pigs per litter per year since 2001 (Figure 1a) [4]. This discrepancy between the yearly increase in litter size at birth versus at weaning could be attributed to the approximately 4% increase in preweaning mortality across the same time period (Figure 1b) [3]. As a result, there are concerns that these genetic improvements may lead to increased tissue mobilization and decreased sow reproductive performance, especially if dietary nutrients are limiting [5]. Therefore, it is generally agreed that the current feeding programs are not providing sufficient amounts of essential nutrients, primarily amino acids, and should be reevaluated to support the improved reproductive performance of modern sows.

In addition to improvements in reproductive performance, the characteristics of moderns sows have changed in regards to leanness and body weight [6]. This change in body composition, in addition to the fact that lactating sows frequently exhibit a decrease in voluntary feed intake, further contributes to nutrient deficiencies and nutrient mobilization during the reproductive period [7]. If highly prolific modern sows are not provided sufficient nutrients in the diet, the sow will mobilize her own body tissues to support reproduction [8], which can result in adverse physiological effects that threaten reproductive performance and longevity [9], and limit the growth and impair the health of offspring [10]. Similarly, it has been established that pre-weaning mortality is increased in piglets born to larger litters [11,12]. This could indicate that highly prolific sows may struggle to support larger litters, either during gestation or lactation.

Researchers have shown interest in investigating the physiological challenges associated with increased reproductive performance and the use of select bioactive compounds to mitigate these effects. Considering the relationship between the sow and their litter, the growth and health of both piglets and sows may be influenced by the use of bioactive compounds during gestation and lactation, including, but not limited to, enzymes, organic minerals, single-cell organisms, and plant oils and extracts [13,14,15]. Therefore, there is a growing need to understand the nutritional challenges of highly prolific modern sows related to improved reproductive performance through gestation and lactation. The objective of this review is to investigate the amino acid requirements of modern sows, in addition to the physiological challenges and the potential effects of select bioactive compounds to improve sow and piglet performance.

## 2. Nutritional Challenges and Opportunities to Update Feeding Programs

The improved reproductive performance of modern sows indicates that current feeding programs may be deficient in some nutrients. Published in 2012, the most recent publication of the *Nutrient Requirements of Swine* (*NRC*) [17] recommends increasing daily feed allowance from day 90 of gestation to farrowing by about 400 g per day, maintaining the same net energy (NE) content of the diet, and increasing the amino acid provision. Commercially, many systems feed sows a single gestation diet and feed allowance is scaled based on visual body condition and stage of gestation. This practice, known as bump feeding, is meant to account for progressing amino acid and energy needs throughout gestation [18]. As the amino acid requirement for gestating sows is dictated in part by the number of fetuses, it would be suggested that the most recent publication of the *NRC* [17] recommends a feeding program based on the litter size and piglet birthweight at the time of publication.

In early gestation, dietary nutrients are primarily contributing to maintenance and recovery of the maternal body, with minimal contributions to fetal development. Throughout mid and late gestation, dietary and maternal contributions to fetal development significantly increase. McPherson et al. [19] demonstrated that protein accretion in fetuses significantly increases after day 70 of gestation, with protein needs increasing more than 18-fold towards the end of gestation. Similarly, Feyera and Theil [20] estimated that the SID lysine requirements would increase by 60% from day 104 to day 115 of gestation suggesting that the current feeding programs would be severely deficient in amino acids in the last days of gestation, even with the utilization of bump feeding. By supplying an increased amount of the same diet, sows will receive excess energy and insufficient amino acids, as pigs eat until they have met their energy requirements [21]. Amino acid deficiency towards the end of gestation can result in poorer litter outcomes and adverse effects on sow longevity and reproductive performance [22]. Increasing amino acid supply should not be carried out by simply increasing feed allowance as it increases energy intake as well, negatively affecting mammary gland growth, milk production, and voluntary feed intake during lactation [23].

In contrast, phase feeding, in which the amino acid-to-energy ratio is based on phase of gestation, could be a more effective feeding program for modern sows should amino acid requirements throughout gestation be well established [24,25]. Phase feeding has historically been limited due to the logistics of commercial pig facilities [26]; however, phase feeding technology is becoming increasingly more available to pig producers. The use of phase feeding and similar precision feeding technologies can also benefit the environment by reducing nitrogen excretion, which is of significant interest to the pig industry. Improved manufacturing processes have improved the availability of crystalline amino acids, allowing for a reduction in dietary crude protein, and reducing waste due to the high bioavailability of crystalline amino acids when compared with traditional protein supplements [27,28]. Regardless of feeding programs employed, establishing updated amino acid requirements will prove useful in improving sow and piglet performance and sow longevity. The following sections will discuss estimated amino acid requirements based on reproductive performance parameters and the impacts of increasing amino acid requirements beyond those of the *NRC*, as investigated in recent literature.

### 2.1. Estimated Amino Acid Requirements Based on Litter Size and Piglet Birthweight

As previously discussed, the increase in both litter size and piglet birthweight will contribute to increased amino acid requirements during gestation. According to Feyera and Theil [20], the standardized ileal digestible (SID) lysine requirement in late gestation is primarily represented by fetal growth (22.7%), mammary growth (16.8%), and colostrum production (16.1%), with the remaining requirement contributing to sow maintenance and uterine development. Kim et al. [25] evaluated the deposition of amino acids in fetal and mammary tissue throughout gestation in order to establish the ideal amino acid balance for sows. Data from this paper were adapted to estimate the change in amino acid requirements during gestation resulting from the increased litter size and piglet birthweight occurring since publication of the *NRC* in 2012 [17]. The lysine gain in fetal tissue was reported to be 0.283 g of lysine per day per fetus [25]. Utilizing the average piglet birthweight suggested by the *NRC* (1.4 kg), the gain in grams per day per fetus could be scaled according to the recent increase in piglet birthweight. The Pig Improvement Company (PIC) reports that piglet birthweight has increased by approximately 0.22 kg per piglet from 2012 to 2022 on their elite farms [29]. Using this, combined with the previously discussed litter size data from PigCHAMP [3], the increase in amino acid requirements per day could be estimated based on increasing litter size and piglet birthweight.

**Figure 2:**
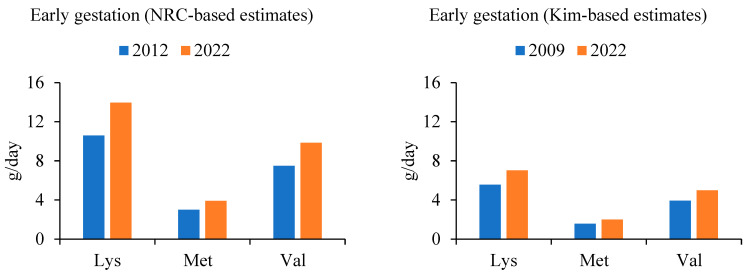
Change in lysine (Lys), methionine (Met), and valine (Val) as dictated by increasing litter size (weight and number of fetuses) as calculated according to the *NRC* model [17] and data from Kim et al. [25], for early gestation (approximately 0 to 70 days gestation).

**Figure 3:**
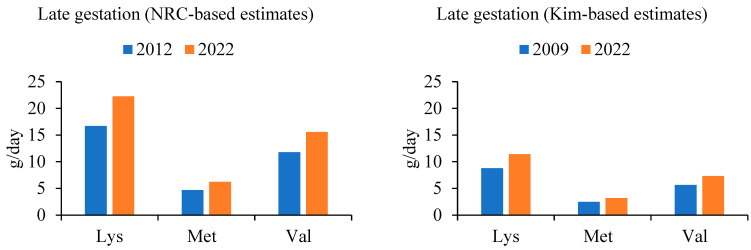
Change in lysine (Lys), methionine (Met), and valine (Val) as dictated by increasing litter size (weight and number of fetuses) as calculated according to the *NRC* model-based estimate [17] and data from Kim et al. [25], for late gestation (approximately 70 days gestation to farrowing).

**Figure 4:**
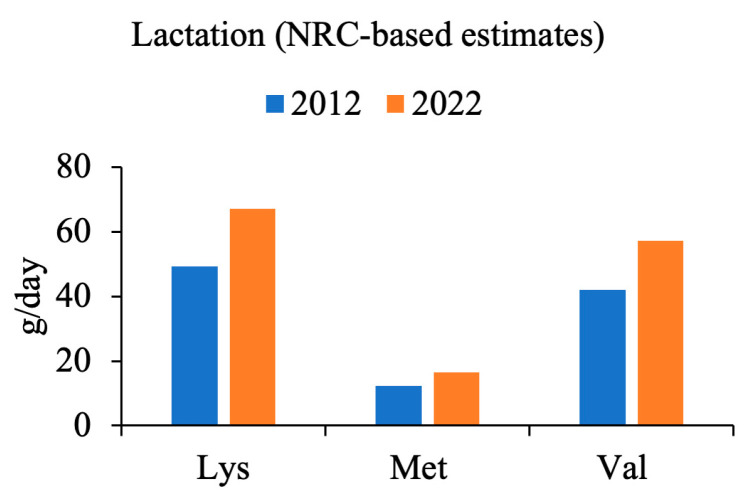
Change in lysine (Lys), methionine (Met), and valine (Val) as dictated by increasing litter size (weight and number of fetuses) as calculated according to the *NRC* model [17], for lactation. No data included regarding lactation in Kim et al. [25].

Two sets of estimates were generated (Figure 2, Figure 3 and Figure 4), both utilizing the maintenance values for lysine in early and late gestation provided by Kim et al. [25]. A ratio was established demonstrating the increase in total litter weight at birth, accounting for both the increase in number of piglets per litter and the increase in individual piglet birthweight. For each estimate generated, amino acid requirements for sow maintenance remained the same, regardless of the year. Amino acid requirements for fetal growth were scaled by year, according to the generated ratio. The Kim-based estimates utilized the analyzed values provided in the literature [25]. Two sets of estimates were created to account for the difference in the initial amino acid recommendations from both sources. The result reveals an estimated 31% (*NRC*-based estimates) and 26% (Kim-based estimates) increase in grams per day of lysine during early gestation, and an estimated 33% (*NRC*-based estimates) and 29% (Kim-based estimates) increase in grams per day of lysine during late gestation, when compared with the current *NRC* feeding program, respectively. Methionine and valine were scaled on a ratio to lysine as reported in their respective sources. Lactation values were not reported by Kim et al. [25]; however, a similar method was applied to calculate the change in amino acid requirements during lactation, compared to those of the *NRC* (Figure 4). For the *NRC*-based estimates during lactation, a 36% increase in lysine requirement was observed.

The recently published *Brazilian Tables for Poultry and Swine* [30] provides an updated feeding program that does show an increase in amino acids in g/day when compared to the *NRC*. Comparatively, the total lysine in g/day for primiparous sows was 16% and 10% greater than the *NRC* total lysine in g/day in early and late gestation, respectively. The estimation featured in the *Brazilian Tables for Poultry and Swine* factors in average body weight, maternal weight gain, and reproductive weight gain. When compared with the models generated for this review, all models recommend an increase in the amino acid provision when compared with the current feeding program proposed by the *NRC*.

### 2.2. Increased Provision of Other Amino Acids

In addition to updating the general feeding program for modern sows, research has shown that adjustment of the amino acid ratios relative to lysine can have additional positive effects on reproductive performance and litter outcomes for modern sows (Table 1). Arginine is a conditionally essential amino acid, and it has been established that arginine can be limiting during gestation and lactation, and for piglets. Due to its status as conditionally essential, no optimal inclusion level has been established; however, it is generally agreed that typical feeding programs suggest insufficient arginine to maintain maximum fetal growth and arginine in sows’ milk is insufficient to support the rapid growth of suckling piglets [31,32]. Early work by Mateo et al. [33,34] demonstrated the functional role of arginine in enhancing reproductive performance of gilts when supplemented in the diets. This initial research supplemented high levels of arginine (0.8% of L-arginine or 1.0% L-arginine HCl) to prove if arginine provides functional benefits. This was further investigated by later studies at lower levels of arginine supplementation. The supplementation of an arginine top-dress at approximately 1.0% of the diet during early gestation was shown to increase the number and total weight of viable fetuses by 39.8% and 32.1%, respectively, when compared to sows fed a traditional gestation diet [35]. Interestingly, Hines et al. [36] investigated the timing and the inclusion level of arginine supplementation during gestation and found that sows provided an additional 1.0% of dietary arginine from day 15 to 45 of gestation had an increased birthweight and preweaning average daily gain when compared to sows provided no additional arginine or 1.0% arginine supplementation beginning on day 45 of gestation. Similarly, Fonseca et al. [37] found that the average weight of piglets born alive was increased by 8.1% in sows fed a diet top-dressed with an additional 1.0% L-arginine from days 30 to 60 and from day 80 to farrowing, when compared with sows not provided additional dietary arginine. The observed increases in reproductive performance relative to increased dietary arginine could be explained by the role arginine serves as a substrate for angiogenesis and vascularization of the placenta [38] and to enhance hyperplasia and hypertrophy of muscles of the developing offspring [39]. Despite this, there is still conflicting evidence on whether supplemental arginine has a significant impact on reproductive performance and offspring viability. For example, a study supplementing gilts with 1.0% of dietary arginine showed no improvements in litter outcomes or gilt retention and future reproductive performance [40]. Similarly, Quesnel et al. [41] investigated the use of an L-arginine supplement at 0.77% of the diet the beginning of day 77 of gestation to farrowing, and there were no effects on sow or litter outcomes, aside from reduced within-litter variation in the sows’ supplements with L-arginine. Notably, one such study found that supplementation of 0.8% dietary arginine from day 0 to 25 of gestation actually decreased uterine weight by 20%, total number of fetuses by 24%, total weight of fetuses by 34%, and total volume of allantoic and amniotic fluids by 34% to 42%, when compared with the control group [42]. As such, the effects of increased dietary arginine as it relates to reproductive performance warrants further investigation.

Similarly, methionine plays a crucial role in fetal growth as it is involved in one-carbon metabolism and serves as a precursor for molecules essential for fetal development and nutrient transport [43]. Furthermore, increases in the Met:Lys ratio above those found in current feeding programs, a Met:Lys ratio of 0.37, have improved vascular density of the sow placenta and increased piglet birthweight [44]. Studies have shown that increasing the Met:Lys ratio to 0.42 in the diets of gestating sows resulted in an improvement in birthweight of piglets and the Met:Lys ratio of 0.52 increased survival rates of piglets [45]. Additionally, the increasing values of methionine alleviated local inflammation by changing the microbial composition of the hosts’ intestinal bacteria and increasing placental vascular density [44,46].

Leucine is known to be a regulator of protein synthesis, namely through its interaction with the mTOR pathway [47]. Leucine is found in the second-highest concentration of all amino acids in the porcine placenta, and promotes protein synthesis in both placental and fetal tissue [48]. One study investigated the use of additional dietary leucine from day 70 of gestation to farrowing to determine effects on piglet growth performance [49]. In this study, a Leu:Lys ratio of 2.65 was demonstrated to improve mean piglet birthweight by approximately 4%, and increased expression of amino acid transporters in the sow placenta in all groups provided additional dietary leucine [49]. These results could be attributed to the role of leucine in the stimulation of muscle protein synthesis and maternal–fetus nutrient transport [47,50,51] and the improved transportation of amino acids, fatty acids, and glucose across the placenta [40].

Primarily for lactating sows, valine is considered to be the second or third limiting amino acid, due to its role in mammary gland metabolism [24], and it plays a significant role alongside the other branched chain amino acids in determining the milk fat content [52]. Research by Che et al. [53] revealed that inclusion of Val:Lys ratio to 0.93 in diets of gestating gilts increased piglet weight at weaning by about 10% and ADG during lactation by 12%, and enhanced protein and fat composition in colostrum and milk, when compared with diets containing a Val:Lys of 0.63 and 0.73. Wang et al. [54] and Zhao et al. [55] also suggested that a Val:Lys ratio between 0.99 and 1.01 may have positive results, improving growth performance of piglets, increasing placental area, increasing lactose concentration in colostrum and serum immunoglobulins in piglets, and decreasing the number of stillborn piglets.

Collectively, these findings demonstrate that increased provision of selected amino acids may have positive impacts on the reproductive performance of pregnant sows and can improve the viability of piglets. Based on literature review, currently there are gaps in research evaluating the requirements of isoleucine and histidine or their optimal ratio to lysine in sow diets. Considering exceptionally high leucine in typical corn soybean meal-based sow diets and ideal balance among branch chain amino acids [56,57], investigation of optimal Ile:Lys ratios and Ile:Val:Leu in sow diets would refine the current feeding program of sows. Considering the functional role of histidine with antioxidative property and behavioral influences through carnosine and histamine [58,59,60] and increased leanness and milk production [2,6] as well as behavioral concerns due to gestation housing [61,62] of modern sows, investigation of the optimal His:Lys ratios in sow diets would refine the current feeding program as well. Thus, careful reexamination of the amino acid requirements for sows could reveal optimal levels that differ from current recommendations.

**Table 1:** Summary of the effects of increasing the provision of arginine (Arg), methionine (Met), leucine (Leu), and valine (Val) relative to lysine (Lys).

Amino Acid	Ratio to Lys	Effect	Reference
Arg:Lys	2.9 (gestation), 2.1 (lactation)	Increased individual weight of piglets born alive by 8% and number of ‘heavy’ piglets	[37]
	1.0	Increased number of live births by 13%	[63]
	1.5	Increased the number of piglets born per sow by 7%, tended to reduce piglet mortality without modifying the sow intestinal microbial structure and gut eubiosis, and increased the placenta weight	[64]
Met:Lys	0.42	Enhanced reproductive performance of sows, alleviated local inflammation by changing the microbial composition of the hosts’ intestinal bacteria	[45]
	0.52	Improved the survival rate of piglets	[45]
	0.37	Increased alive litter weight by 8% for high-prolificacy sows	[44]
	0.37	Decreased plasma homocysteine concentration, allowing the increase in placental vascular density	[46]
	0.42	Increased the antioxidant capacity and improved the intestinal microbiota in piglets	[65]
Leu:Lys	2.15 and 2.65	Improved the transportation of amino acids, fatty acids, and glucose across the placenta, and globally altered placental metabolism to enhance glycolysis and fatty acid oxidization for energy generation	[51]
	2.65	Improved growth performance of fetal pigs	[49]
	2.15, 2.65, and 3.16	Increased expression of amino acid transporters in the sow placenta	[49]
Val:Lys	0.99 and 1.11	Increased placental area, decrease stillborn, and improve pig performance at weaned period, by increasing the amino acid and glucose transports	[54]
	0.93	Improved piglet weaning weight by 12% and ADG during lactation by 13%	[53]
	1.01	Improved the growth performance of piglets by altering serum metabolites in sows, the lactose concentration in colostrum, and serum immunoglobins in piglets	[55]

## 3. Physiological Challenges and Opportunities with Bioactive Compounds

As previously discussed, modern sows face unique challenges resulting from increased reproductive performance, which can sometimes result in adverse physiological effects that threaten sow performance and longevity [9]. In some cases, these physiological effects can also negatively influence the piglets. To mitigate these physiological effects, the use of select bioactive compounds have been investigated in sows with varying success and have the potential to improve sow and piglet outcomes. A summary of the adverse physiological effects and research regarding opportunities for the use of select bioactive compounds can be found in Table 2.

### 3.1. Oxidative Stress

Oxidative stress occurs when the generation and accumulation of reactive oxygen species (ROS) exceeds the ability to detoxify and remove these products from body tissues [82]. Reproduction causes sows to be in a constant state of oxidative stress. During gestation, the placenta produces ROS like superoxide and hydrogen peroxide, and a significant drop in antioxidants in circulation can be observed as gestation progresses, specifically retinol and α-tocopherol [83,84]. The placenta does have the ability to produce antioxidative enzymes and hormones to mitigate excessive lipid peroxidation [85], but still, oxidative stress in sows is unavoidable due to the high metabolic demands of reproduction [62,86].

Although oxidative stress related to reproduction is normal, the inability to reduce and recover from oxidative stress after farrowing creates physiological imbalances that can cause postpartum dysgalactia syndrome (PDS), the most common disease to affect sows after parturition [87]. Postpartum dysgalactia syndrome is a complex disease that is primarily associated with the symptoms of mastitis and metritis, and has become the more widely accepted name for a previously used term, mastitis-metritis-agalactia (MMA) [88]. Postpartum dysgalactia syndrome has been reported to affect as much as 37% in some modern sow herds and incidence within the herd is thought to be underestimated due to the wide range of clinical symptoms associated with the syndrome [89,90]. In particular, PDS has received attention due to its role in lactation failure, typically related to mastitis resulting in sow culling [91]. Although it is often unclear whether oxidative stress or PDS induce one another, respectively, it is understood that they often occur in conjunction, with oxidative stress markers like 8-epi-PGF2α being increased in sows with PDS [92]. Berchieri-Ronchi et al. [83] demonstrated the oxidative damage associated with reproduction by measuring DNA damage throughout gestation and lactation, finding that DNA damage significantly increased from day 60 of gestation and did not resolve until the weaning period. Similarly, Lin et al. [84] showed that systemic oxidative stress immediately following farrowing, combined with impaired immune function, makes the sows more vulnerable to mastitis. In this study, serum malondialdehyde (MDA) concentration was found to be higher at 28 days post-parturition than at 90 days of gestation and α-tocopherol was decreased at parturition when compared with both day 90 of gestation and at day 28 post-parturition. Similarly, a down-regulation of the pro-inflammatory cytokines in mammary tissue was observed surrounding parturition, with a tendency for the expression of these cytokines to increase throughout the lactation period. This result can be explained by the fact that mammals will go through a period of immunosuppression in the periparturient period to protect the fetus(es) from potential harm by the maternal immune system [93], which can ultimately have negative effects on the health of the mother.

The inclusion of select dietary bioactive compounds in feeds could be an effective way to reduce oxidative stress during gestation and lactation. Bioactive compounds such as phenols and polyphenols, flavonoids, vitamin C, vitamin E, beta-carotene, zinc, and selenium have been shown to have antioxidant properties and the ability to scavenge free radicals [94,95]. In particular, selenium, vitamin E, and vitamin C have been investigated for their ability to reduce oxidative stress and associated inflammation [96] and much research has been carried out regarding the use of these compounds when pigs may be at risk of increased oxidative stress, such as at weaning or during gestation and lactation [96,97,98,99,100]. Typically, plant-derived compounds with antioxidant properties are present in small amounts. As such, high doses of refined bioactive compounds, such as oils or extracts, are often utilized to achieve maximum antioxidant function [101].

To reduce oxidative stress, two different studies demonstrated that the antioxidant status of gestating sows could be improved by the dietary inclusion of organic selenium, evidenced by a decreased MDA content and an increased total antioxidant capacity in serum [66,67]. Additionally, many plant-derived compounds have been shown to have positive effects on sow antioxidant status and reproductive performance. Catechins, a plant-derived polyphenolic compound, included in the diet at 200 and 300 mg/kg, increased the number of piglets born alive per litter, increased the number of healthy piglets born per litter, and decreased the stillborn rate [68]. Similarly, the inclusion of Moringa oleifera at 4% and 8% of the diet was shown to significantly decrease the length of farrowing, decrease the number of stillborn, and tended to increase the number of piglets born alive, while also showing a decrease in sow serum MDA [69]. Polyphenols derived from grape seed included at 200 to 300 mg/kg of the diet exhibited positive effects on the activity of superoxide dismutase and glutathione peroxidase, as well as increased the immunoglobulin levels in colostrum [71]. Finally, oregano essential oil included at 15 mg/kg of the diet was shown to reduce the serum concentration of 8-hydroxy-deoxyguanosine (8-OHdG) and thiobarbituric acid reactive substances (TBARS) on day 1 of lactation, increase sow feed intake during gestation, and increase average daily gain of piglets [70].

### 3.2. Prolapse and Lameness

Sows must remain in the herd for three or more parities to offset the cost of raising, feeding, and maintaining the breeding sow; therefore, efforts should be made to address any limitations to longevity [9]. According to the results of a survey-based project published in 2019, approximately 21% of all sow mortalities can be attributed to pelvic organ prolapse (POP) [102]. For many years, researchers have attempted to pinpoint risk factors associated with pelvic organ prolapse, but the reasons behind POP are multifactorial and cannot be attributed to a specific risk factor. Most POP will occur surrounding farrowing, with 60% of prolapse removals occurring during the first 0 to 4 weeks after farrowing [103]. Furthermore, sows with a greater number of stillborn piglets are more likely to experience pelvic organ prolapse, but this correlation may be explained by the increase in stillborn piglets associated with difficult farrowing, such as a large time interval between births and large piglet birthweight or litter size, which may inadvertently cause pelvic organ prolapse [1]. Finally, it is thought that generalized stress, such as pen versus group housing or excessive metabolic burden associated with large litters, can increase the risk of pelvic organ prolapse [61,104].

One common dietary strategy to reduce the risk of pelvic organ prolapse is to increase the fiber content in the diets of sows close to farrowing. Constipation commonly occurs in sows near the end of gestation, typically due to both limited feed intake and decreased intestinal motility surrounding farrowing [72,105]. Feces that have yet to be excreted can act as a physical barrier to the birthing canal and prolong farrowing, as demonstrated by Oliviero et al. [106], where it was identified that increasing constipation was positively correlated with an increase in farrowing duration. It was found that the inclusion of 7% crude fiber in the 5 days before and after farrowing reduced constipation and prompted a faster return to normal intestinal activity when compared with sows fed a diet containing 3.8% crude fiber; in addition, increasing the dietary crude fiber also increased the average daily water intake, which can aid in relieving constipation and increase milk yield during lactation [72].

Lameness is another significant concern for breeding sows as it decreases their lifetime productivity and overall welfare. Some common causes of sow lameness include osteochondrosis (OCD), arthritis, claw lesions, and injuries. Similar to pelvic organ prolapse, the reasons for sow lameness typically cannot be attributed to a single risk factor [107]. The incidence of OCD has increased with the introduction of new genetics exhibiting accelerated growth rates and can be exacerbated by an imbalance or deficiency in dietary vitamins and minerals. Primarily, OCD affects gilts as the onset of disease occurs during growth and causes bone and cartilage lesions that present during skeletal growth and maturation [108].

Generalized lameness can be a result of poor weight management during gilt development or a vitamin or mineral deficiency. Lactating animals producing large quantities of milk are at risk of calcium (Ca) and phosphorus (P) depletion of the bone reserves which can leave the sow at higher risk of injury and lameness [109,110]. In addition, biotin is necessary for maintaining the strength of the hoof horn and vitamin D plays an important role in Ca and P absorption, all of which are needed to maintain bone health and strength [111,112]. Phytase is commonly utilized in feeds to increase Ca and P availability in feeds and can reduce the need for Ca and P supplementation from sources like limestone and dicalcium phosphate [113]. However, it is important to consider the Ca to P ratio when increasing Ca content to support smooth muscle function and skeletal integrity. Excessive Ca can decrease the sow’s ability to utilize P or other minerals, like magnesium, and may require additional mineral supplementation to prevent Ca antagonism [114,115]. In addition to Ca and P, the dietary inclusion of additional minerals could support soundness in sows. For example, the use of organic minerals (10, 20, and 50 mg/kg of organic Cu, Mn, and Zn, respectively) during the rearing phase for gilts significantly decreased the incidence of lameness when compared with gilts fed a typical diet during the rearing phase [73]. Incidence of lameness was reduced by mineral supplementation during rearing by 12.8% and 14.3%, during the rearing phase and first lactation, respectively [73]. Similarly, another study found that partial substitution of inorganic salt forms of minerals with organic trace minerals (Zn, Cu, Mn) reduced the odds of a higher versus lower lesion score when compared to sows provided a diet consisting of minerals exclusively in their inorganic salt form [74]. It is understood that organic sources of trace minerals are more stable and less likely to form inaccessible compounds with other nutrients, and therefore, may be more bioavailable to the animal, thereby increasing the effects associated with increased mineral supplementation on sow lameness [116].

### 3.3. Colostrum and Milk Production

During lactation, sows must produce enough high-quality colostrum and milk to support the immune development and growth of the litter. Despite increases in litter size, colostrum and milk yield have not increased at the same rate over the last 30 years, indicating that increases in milk production have not effectively followed the increase in litter size [117]. It has been demonstrated that litter size does not influence colostrum yield, indicating that piglets born to large litters may be at a disadvantage compared to piglets born to smaller litters [118]. In contrast, milk production has been shown to be positively correlated with increasing litter size, suckling intensity, and mammary gland location [119,120,121,122,123]. Similarly, it is known that the milk yield differs for each gland, with the anterior glands being more productive than the posterior glands [124], and teats that are not suckled will become involuted and will stop producing milk for the current lactation and can have impacts on subsequent lactations [125]. In regards to composition, there is some evidence that milk protein may be decreased with increasing litter size during late lactation; however, this study did not utilize modern genetics or rearing techniques, as piglets were not weaned until 28 days; therefore, the relationship between litter size and milk composition is a topic in need of further investigation [122]. Nonetheless, the strong positive correlation between milk yield and increasing litter size leaves many concerned with the potential negative effects of large litters, as heavy milk production is often associated with high rates of maternal tissue mobilization, which may negatively affect the development and maintenance of mammary tissue in the current lactation, as well as impact sow longevity and future reproductive performance [126,127].

Due to the physiology of the porcine placenta, piglets are unable to acquire maternal immunoglobulins during gestation [128]. Circulating immunoglobulins acquired from sow colostrum and milk serve as passive immunity as the first line of defense during the development of the adaptive immune system. Because of the importance of maternal immunoglobulin transfer to the litter and concerns surrounding the increased competition for maternal colostrum, opportunities to increase immunoglobulin concentrations are of particular interest for researchers. The most common immunoglobulins are IgG, IgA, and IgM, with IgG being the most prevalent immunoglobulin in colostrum at a relative content of approximately 75%, and IgA being the most prevalent immunoglobulin in milk, at a relative content ranging from 50 to 60% throughout lactation [129]. A study utilizing radioactive labeling of sow immunoglobulins determined that IgG, IgA, and IgM are derived from sow serum at rates of 100, 40, and 85%, respectively [130]. The remaining IgA and IgM present in milk are produced locally by plasma cells that have migrated into the mammary gland [131,132]. Typically sows will have a greater immunoglobulin content in milk when compared with gilts, meaning piglets born to sows will have a greater chance for absorption of immunoglobulins from colostrum [133]. It has been established that the efficiency of IgG uptake by piglets is approximately 25.2% to 28.5% from sow colostrum [134,135] and that the true digestibility of proteins and the dry matter of colostrum is nearly 100% [135].

Several bioactive compounds from feedstuffs can trigger an immune response and enhance the immunoglobulins present in colostrum and milk. Polysaccharides derived from plants, seaweeds, and yeasts have the potential to influence the immune system. For example, polysaccharides extracted from the seaweed Ulva armoricana and from the herb Astragalus mongholicus have been shown to induce an immune response in sows, thus increasing immunoglobulin concentrations in sow colostrum and milk [75,76]. In addition, polysaccharides from the yeast cell wall, mannan oligosaccharides, have received attention due to their potential interactions with the immune system, and thereby growth performance, as first investigated in nursery pigs [136,137], as bioactive compounds that can induce a maternal immune response that could be transferred through colostrum or milk [138]. Davies et al. [137] reported that dietary supplementation of mannans derived from the yeast cell wall stimulated the immune system of nursery pigs by positively influencing the T lymphocyte production of the jejunal lamina propria, and this localized immune response can trigger a systemic immune response in the host. Yeast-based feed additives are known to have a positive impact on the immune response that could benefit piglets as they face challenges associated with disease exposure and weaning stress, should these effects be transferred from the sow to the offspring [77,78]. Research has shown that yeast-based feed additives enhanced the overall reproductive performance of sows [139,140,141,142], perhaps through enhanced immune system activation contributing to improved outcomes. The controlled use of bioactive compounds as immune system stimulators could serve a similar role as vaccine protocols in gestating sows, and there are some instances of plant-derived compounds being utilized as vaccines for specific pathogens [143,144]. However, immune activation resulting from dietary yeast-based additives will be more general whereas vaccine protocols and plant-derived vaccines will induce a specific immune response to a pathogen or common class of pathogens.

In addition to immunoglobulins, sow colostrum and milk are relatively high in fat, which can benefit piglets by supporting the energy system for body homeostasis and maintenance during early life [145]. Increasing fat in maternal diets could increase milk fat concentrations, improve piglet weight gain, and provide the sow with additional energy to reduce excessive weight loss during lactation [146]. Larger litters will have increased competition for colostrum and milk; therefore, increasing the energy content of colostrum and milk could potentially benefit rapidly growing piglets. Researchers found that soybean oil included at 3% of the diet for the week prior to farrowing increased colostrum fat concentrations and enhanced immunoglobulin plasma levels in both sows and piglets when compared to the other fat sources studied, coconut and palm oil [147]. Similarly, Bontempo et al. [148] found that maternal dietary supplementation with conjugated linoleic acid (CLA) increased circulating serum IgG in sows, increased IgG concentrations in colostrum, and increased serum IgG in piglets. Conjugated linoleic acid is known to have a multitude of positive effects on growth and the immune system, as reviewed by Pariza et al. [149]. In general, functional fatty acids, such as CLA and linoleic acid, can improve piglet vigor by providing highly digestible energy, and having functional effects such as anti-inflammatory and immunomodulatory properties.

### 3.4. Intestinal Health

Positive modulation of the mucosa-associated microbiome through the use of bioactive compounds can assist in mitigating oxidative stress and general inflammation, a challenge for highly prolific sows. A study by Tan et al. [79] demonstrated that fiber has the potential to encourage the proliferation of specific bacterial populations that can reduce oxidative stress. In this study, between a control group of sows and a group of sows being fed a basal diet with an inclusion of 2.2% konjac flour (KF), sows in the KF group exhibited a decrease in the relative abundance of Proteobacteria in the fecal microbiota, a phylum commonly associated with intestinal inflammation, and a reduced serum level of ROS [150]. Alternatively, the dietary inclusion of 1.6% inulin, a soluble dietary fiber, resulted in the favorable Firmicutes to Bacteriodes ratio in the feces of sows when compared to sows not fed diets containing inulin [80]. As a result, reproductive performance was improved, including decreasing piglet diarrhea, improving litter weight gain, survival rate, and average daily gain during lactation, and a significant linear increase in serum superoxide dismutase and glutathione peroxidase, important enzymes aiding in the antioxidant capacity of cells, was observed, as the inclusion level of dietary inulin was increased [80].

Probiotics can also have a positive impact on oxidative stress by encouraging the growth of beneficial microbial populations and inhibiting the growth of harmful microbial populations of the large intestine. In a study by Hayakawa et al. [81], the inclusion of probiotics from three different sources (*Bacillus mesentericus*, *Clostriduim butyricum*, and *Enterococcus faecailis*) in sow diets was found to shorten the length of gestation and increase litter weight at delivery, although no improvements were seen in the growth parameters of piglets suckling from sows fed the probiotics. Similarly, some studies have found evidence that maternal probiotic supplementation can increase the populations of potentially beneficial microbial populations in the feces of both sows and piglets [151,152,153] and a reduction in populations of potentially harmful bacterial populations, like the reduction of β-haemolytic *E. coli* isolates in piglet feces nursing from sows fed a probiotic during gestation [154], all of which can improve piglet growth performance and health.

Development of the microbiota of the gastrointestinal tract during the suckling period can have a significant impact on intestinal health and the intestinal immune system at weaning. It is still unclear whether the first intestinal exposure to bacteria occurs in utero or during parturition [155,156,157]. However, it is well known that farrowing and subsequent exposure to the environment have a significant influence on the piglet microbiota in the small and large intestine [158]. The predominant phyla present vary the length of the gastrointestinal tract, with Proteobacteria (76.0%) and Firmicutes (22.2%) being most abundant in the small intestine, and Firmicutes (78.3%) and Proteobacteria (13.0%) being most abundant in the large intestine [159]. Throughout suckling, the predominant genus found in piglet feces is Bacteroides, which gives way to *Prevotella* spp. after weaning [159,160,161]. Bacteroides have the ability to utilize most of the milk oligosaccharides (MOS) found in sow milk [162], which make up a significant portion of mammalian milk [163]. Large MOS are not absorbed in the gastrointestinal tract, allowing for microbial fermentation of MOS, which can encourage the proliferation of potentially beneficial bacteria like Bifidobacterium and *Lactobacillus* sp. [164]. Research suggests that supplementation of Bifidobacterium species may improve gut health and reduce the effects of diarrhea in challenged weaned pigs, possibly through the production of short-chain fatty acids, such as acetate [165,166,167]. Most research regarding the effects of MOS on the intestinal health of suckling piglets has been conducted using milk replacer, fortified with human or bovine MOS [168,169,170,171]. As such, it would be worth investigating maternal nutritional interventions to increase the amount of MOS in sows’ colostrum and milk to emulate these effects, especially for large litters that may have an overall decreased milk consumption due to competition.

## 4. Conclusions

Rapid genetic advancements have significantly enhanced the reproductive performance of modern sows. However, these improvements come with nutritional challenges, particularly in managing the increased metabolic burden and the associated adverse physiological effects. These adverse physiological effects can also have implications for piglet survivability, perinatal mortality, and litter performance. Current feeding programs may be insufficient to support the nutritional needs of the modern sow, and updated research is necessary to establish an appropriate feeding program based on the increased reproductive performance. In addition to the nutritional needs of the modern sow, the use of bioactive compounds can possibly improve reproductive performance and enhance the growth and health of suckling piglets. The use of additional mineral supplementation, enzymes, algae and yeast-based derivatives, and fiber sources and probiotics all have the potential to reduce the impact of adverse physiological effects caused by enhanced reproductive performance. In conclusion, thoughtful nutritional management strategies are crucial to address these concerns and ensure the well-being and performance of both sows and piglets.

## Figures and Tables

**Table 2:** Summary of nutritional interventions to mitigate adverse physiological outcomes in modern sows.

Concern	Intervention	Effect	Reference
Oxidative stress	Selenomethionine (0.3 mg Se/kg diet)	Increased litter weaning weight, increased total antioxidant capability, and decreased MDA in sows, increased glutathione peroxidase, superoxide dismutase in colostrum	[66]
	Organic Se (0.3 mg/kg diet)	Increased litter birthweight, greater number of pigs weaned, increased total antioxidant capability, decreased MDA	[67]
	Catechins (200 or 300 mg/kg diet)	Increased litter born alive, litter born healthy, decreased stillborn rate	[68]
	Catechins (100, 200, 300, or 400 mg/kg diet)	Decreased sow serum H_2_O_2_ levels	[68]
	*Moringa oleifera* (4 or 8% of diet)	Reduction in farrowing duration, decrease in number of stillborn, decreased sow serum MDA, increased total antioxidant capability, reduced serum nitrogen in sows and offspring	[69]
	Oregano essential oil (15 mg/kg diet)	Reduced sow serum concentration of 8-hydroxy-deoxyguanosine and thiobarbituric acid reactive substances, increased sow feed intake during lactation, increased average daily gain of piglets	[70]
	Grape seed polyphenols (200 or 300 mg/kg diet)	Increased activity of superoxide dismutase and glutathione peroxidase, higher IgM and IgG content in colostrum	[71]
Prolapse and lameness	Crude fiber (7%)	Increased sow water intake during lactation, decreased constipation and faster recovery to normal intestinal activity post-farrowing, increased piglet weight gain at day 5	[72]
	Organic minerals (10, 20, and 50 mg/kg of Cu, Mn, and Zn, respectively)	Decreased the incidence of lameness during rearing by 12.8% and during lactation by 14.3%	[73]
	Partial substitution of inorganic salts with organic trace minerals (Zn, Cu, Mn)	Reduced the odds of higher versus lower lesion scores	[74]
Colostrum and milk production/quality	Algal sulfated polysaccharide (Ulva aromricana,8 or 16 g/day)	Improved specific IgG transudation from blood to colostrum, increased total IgA titer in milk 7 days post-farrowing	[75]
	Astragalus polysaccharide (1.5 g/day)	Improved levels of IgM and IgG in colostrum 7 days pre-farrowing, increased maternal-derived antibodies to vaccinated diseases in colostrum	[76]
	Yeast-based nucleotide (4 g/day)	Increased litter weaning weight, decreased diarrhea in piglets, improved ileal villus development in piglets, increased expression of interleukin (IL)-17, IL-8, IL-1β, IL-10, and tumor necrosis factor (TNF)-α in the jejunal and ileal tissue of piglets	[77]
	Yeast-derived postbiotic (1.25 or 2.00 g/kg diet)	Increased sow backfat deposition during late gestation, improved IgA, lactose content in milk, greater concentrations of IgG and IgM in piglet serum, decreased piglet diarrhea and mortality	[78]
Intestinal health	Fiber, konjac flour (2.2%)	Decreased serum ROS, increased relative abundance of Proteobacteria in fecal samples	[79]
	Fiber, inulin (1.6%)	Increased serum superoxide dismutase, glutathione peroxidase	[80]
	Probiotics (*Bacillus mesentericus*, *Clostriduim butyricum*, and *Enterococcus faecailis*)	Shortened length of gestation, increased litter weight at birth	[81]

## Data Availability

Not applicable.
